# Who Should Be Considered for Islet Transplantation Alone?

**DOI:** 10.1007/s11892-017-0847-6

**Published:** 2017-03-14

**Authors:** Nantia Othonos, Pratik Choudhary

**Affiliations:** Department of Diabetes, King’s College London, Denmark Hill, London, SE5 9RJ UK

**Keywords:** Type 1 diabetes, Islet cell transplantation, Hypoglycemia, Impaired awareness of hypoglycemia, Severe hypoglycemia, Transplantation

## Abstract

**Purpose of Review:**

Episodic hypoglycemia is an almost inevitable consequence of exogenous insulin treatment of type 1 diabetes, and in up to 30% of patients, this can lead to impaired awareness of hypoglycemia. This predisposes to recurrent severe hypoglycemia and has a huge impact on quality of life. Although many patients can get resolution of severe hypoglycemia through novel education and technology, some patients continue to have ongoing life-threatening hypoglycemia. Islet transplantation offers an alternative therapeutic option for these patients, in whom these conventional approaches have been unsuccessful. This review discusses the selection process of identifying suitable candidates based on recent clinical data.

**Recent Findings:**

Results from studies of islet transplantation suggest the optimal recipient characteristics for successful islet transplantation include age >35 years, insulin requirements <1.0/kg, and weight >85 kg.

**Summary:**

Islet transplantation can completely resolve hypoglycemia and near-normalize glucose levels, achieving insulin independence for a limited period of time in up to 40% of patients. The selection of appropriate candidates, optimizing donor selection, the use of an optimized protocol for islet cell extraction, and immunosuppression therapy have been proved to be the key criteria for a favorable outcome in islet transplantation.

## Introduction

Type 1 diabetes mellitus (T1DM), caused by autoimmune destruction of insulin-producing beta cells, is treated with exogenous insulin therapy. In health, the production of insulin is very controlled, allowing stable glucose values despite food, exercise, or illness. Pharmacological replacement using current tools is very blunt, even in patients using modern technology such as insulin pumps and, certainly, for the majority of patients who use multiple daily injections to try and mimic physiological requirements [1]. They are limited by slow onset and prolonged duration of action of even the most rapid acting insulin analogs used to cover meal time requirements, and peaks and variability in the pharmacokinetics of currently available long-acting insulin analogs required to cover basal requirements. Ongoing management and attainment of glucose levels that reduce the risk of micro- and macro-vascular complications requires frequent glucose monitoring, and multiple decisions every day around food, insulin and activity. This places significant burden on the person with diabetes, often termed diabetes distress and leading to increased rates of depression [2]. It is not surprising that many if not most people with T1DM are unable to achieve optimal glucose control [3]. Given the pharmacological barriers, the risk of striving to minimize high glucose levels is excessive exposure to hypoglycemia. This comes with its own risks including confusion, injury, unconsciousness and even in rare cases death [4]. Repeated exposure to glucose levels below 3 mmol/l can blunt physiological warning signs leading to impaired awareness of hypoglycemia [IAH] that increases the risk of severe hypoglycemia 3–6 fold [5].

In this setting, cellular therapy providing biological insulin replacement therapy for patients with T1DM has been an attractive option for decades. The first reported cases of pancreatic tissue transplants for this purpose where performed by Dr. Frederick Pybus and date back to 1916 [6]. However, it wasn’t until years later, in 1990, when Tzakis et al.[7] reported the first successful islet cell transplantation (ICT) whereby they used an islet isolation method that was first developed by Dr. Paul Lacy [8] and later modified by Dr. Camillo Ricordi [9]. The landmark report of seven consecutive patients achieving insulin independence by Shapiro et al. in 2000 [10], signaled a surge in interest and a number of centers worldwide started offering ICT based on the Edmonton protocol, which included a steroid-free immunosuppression regimen and multiple donor islets. This protocol still forms the basis of ICT protocols used worldwide in islet transplant centers.

Recent data published by the Collaborative Islet Transplant Registry (CITR) showed that the efficacy and safety of this treatment has significantly improved over the last 15 years. While insulin independence is considered to be the primary end-point in most clinical trials of ICTs, it is certainly not the only benefit it offers. The CITR [11••] report indicated that in the presence of even moderate amounts of endogenous C-peptide, there was significant improvement in glycated hemoglobin (HbA1c), which is the crucial factor in reducing the risk of developing chronic complications of diabetes such as those affecting the vasculature and peripheral nerves. More importantly, there was a reduction and in most cases complete abolition of severe hypoglycemic episodes, with resolution of IAH in these patients. Therefore, ICT can offer an alternative form of treatment for patients with T1DM who cannot achieve adequate glycemic control using intensive exogenous insulin treatment and suffer from debilitating recurrent hypoglycemic episodes.

In this review, we will discuss the selection process in identifying suitable ICT recipients based on the current clinical data.

### Current Clinical Data on Islet Cell Transplantation

A key change in emphasis for the outcomes of ICT was triggered by the selection of patients with recurrent severe hypoglycemia in the landmark trial by Shapiro et al.[10]. Other important elements were steroid-free immunosuppression with dacluzimab induction and low-dose tacrolimus and daclizumab for maintenance. In 2006, the Immune Tolerance Network study presented the results of this multicenter phase 1–2 trial, with participants from nine international centers: six in North America and three in Europe, where they investigated the feasibility and reproducibility of the Edmonton protocol across the different centers [12]. Similarly to the aforementioned trial, the eligibility criteria included undetectable C-peptide levels, T1DM for >5 years with recurrent neuroglycopaenia, IAH, and severe glycemic lability and ages between 18 and 65 years old. The islet infusion requirements included the ABO blood group compatibility and an islet mass of ≥5000 IEQs/kg of recipient’s body weight for the initial infusion, with a cumulative mass of over 10,000 IEQ’s delivered with at least two infusions, unless insulin independence was achieved with a single infusion. At 12 months, post final transplantation, 16 of 36 participants (44%) had reached the primary end point, i.e., were insulin independent, 10 participants (28%) had partial graft function, and 10 participants (28%) had complete graft loss.

In 2005, Hering et al. [13] reported the results of a prospective, single-center, 1-year follow-up pilot trial where the primary outcome was insulin independence in the first year after a single-donor islet transplant. Again, this study targeted adult patients with labile glucose levels and IAH. Immunosuppression was more aggressive, with induction using rabbit antithymocyte globulin (rATG), methylprednisolone, daclizumab, and etanercept. Maintenance immunosuppression was initiated with sirolimus and reduced-dose tacrolimus, which was gradually replaced with mycophenolate mofetil one month post-transplantation. All eight recipients became insulin independent and did not experience further hypoglycemic episodes. Of the eight recipients, five remained insulin independent for longer than one year and the rest were insulin-independent for 121, 76, and 7 days. They concluded that contributing factors to their high success rate were excluding pancreata from donors older than 50 years, limiting cold storage to less than 8 h and using the two-layer preservation method, avoiding use of Ficoll during islet purification, and culturing islets pre-transplantation. With regard to immunosuppression, it is possible that etanercept could also contribute to the high success rate, as it was the new addition to this trial compared to the group’s previous trial [14]. In addition, replacing or minimizing tacrolimus at 1 month post-transplantation may have enhanced the function of engrafted islets.

The latest data on ICT are from the Clinical Islet Transplantation (CIT) Consortium study, a phase 3 registration trial, designed to enable licensure of the islet product [15•]. In this study, the participants enrolled were 26.2–65.5 years old, duration of diabetes was 11–57 years, median HbA1c was 7.2% (range, 5.7–9.2) or 55 mmol/mol (range, 39–77), with absent stimulated C-peptide levels, and documented IAH. The exclusion criteria for the study were BMI >30 kg/m^2^, weight ≤50 kg, insulin requirements >1.0 units/kg/day or <15 units/day, HbA1c level >10% (86 mmol/mol), measured GFR <80 mL/min/1.73 m^2^, history of panel reactive anti-HLA antibodies by flow cytometry, and significant comorbidities. Forty-eight subjects were recruited of which 22 subjects received one infusion, 25 received two infusions, and one subject received three infusions. The subjects were given immunosuppression including rATG and etanercept for the first transplant and etanercept with basiliximab at subsequent transplants. Sirolimus and tacrolimus were used for maintenance. The purified human pancreatic islets were manufactured using a standardized method [16]. The primary end point was defined as an HbA1c level of <7.0% (53 mmol/mol) at day 365 with no severe hypoglycemic episodes from day 28 to day 365 after the first islet transplant and was achieved by 42 of the 48 subjects (87.5%). Additionally, 11 subjects achieved insulin independence at day 75, 25, at day 365 and 20 remained insulin independent at day 730. In terms of safety, the authors reported that serious adverse events attributed to the treatment included transplant procedure-related bleeding in 5 of 56 percutaneous cannulations of a portal vein, two infections as a consequence to immunosuppression, and decrease in median GFR. It decreased from 102 ml/min/1.73 m^2^ (range, 80–130) at baseline to 90 ml/min/1.73 m^2^ at day 365 (*P* = 0.0008 vs. baseline, range, 59–129 ml/min/1.73 m^2^).

The CITR collects and publishes data of the islet cell transplant units in North America, Europe, and Australia where the patients have given written informed consent. It is worth noting that in the USA, ICT is experimental but at the Canadian, European, and Australian sites, the treatment is available both as part of a trial or standard of care. In 2013, they published the most recent report which analyzed the data from the patients who received ICT from 1999 to 2012 [11••]. A total of 864 patients received an allogeneic islet transplant of which, 686 were islet transplant alone and 178 following or with a simultaneous kidney transplant, and received a total 1679 infusions from 2146 donors. The recipients were 7–72 years old (mean 45 ± 10SD), had T1DM for 2–61 years (29 ± 11), and had poor glycemic control. Poor glycemic control was defined as frequent episodes of hypoglycemia, severe hypoglycemic episodes, blood glucose lability, and HbA1c >8% [11••]. Over the years, recipient characteristics have been changes in both donor and recipient characteristics. Donors have been getting older with higher BMI, and recipients have also been getting older with higher HbA1c and increased use of continuous subcutaneous insulin infusion (CSII) prior to transplantation.

There has also been a change in outcomes that has been attributed to some extent to change in immunosuppression, with more routine use of T cell depletion with or without TNF antagonists. This has been associated with an increase in 5-year graft survival [11••]. The latest CITR data suggest up to 50% of patient can achieve insulin independence, with over 70% maintaining protection from severe hypoglycemia over 5 years.

### Stepwise Approach to Recurrent Severe Hypoglycemia

The data presented show that while ICT can achieve insulin independence for a proportion of recipients, it is most effective at resolving problematic hypoglycemia. In particular, it is the ability to do this while achieving optimal glucose control. For this reason, in most countries where ICT is available, the main indication is persistent problematic and recurrent episodes of severe hypoglycemia despite optimal medical therapy. Severe hypoglycemia, defined as “an event requiring assistance of another person to actively administer carbohydrates, glucagon, or take other corrective actions,” occurs in around 25% [17] of patients with T1DM and contributes to substantial morbidity [18].

**Fig. 1:**
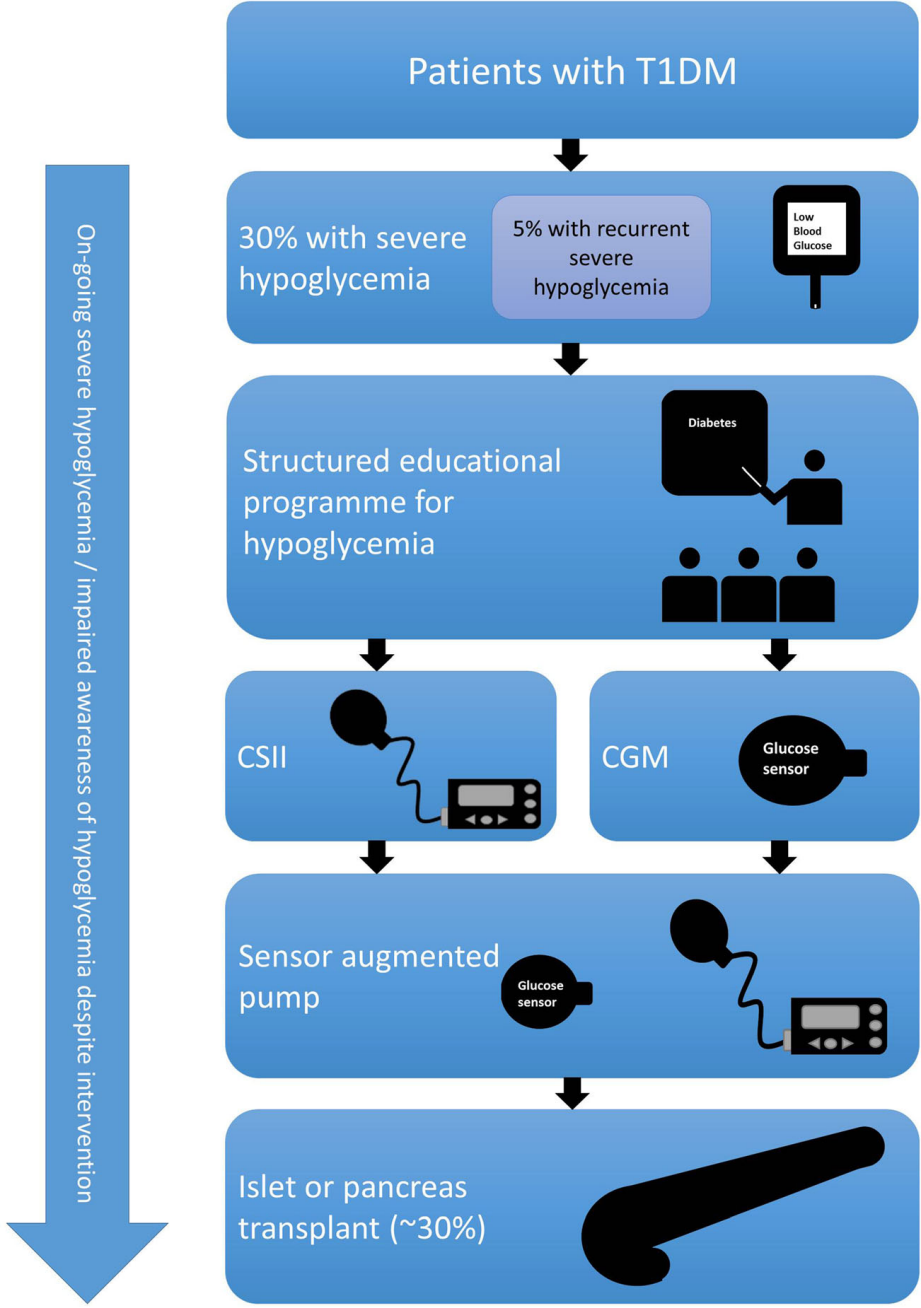
Stepwise approach to recurrent severe hypoglycaemia. *T1DM* type 1 diabetes mellitus, *CSII* continuous subcutaneous insulin infusion, *CGM* continuous glucose monitoring

The first step is identifying patients at high risk of severe hypoglycemic episodes (SHE) (Fig. 1), and the main predictors are duration of diabetes, age, IAH, and previous episode of SHE. For this reason, we advocate routine screening for IAH, just as we would do for microalbuminuria using validated assessment tools that have been shown to predict further episodes of SHE. The most common are the Gold score [19], the Clarke score [20], and the Pedersen-Bjergaard score [21].

Meticulous avoidance of mild hypoglycemia has been shown to restore awareness in experimental settings [22]. In those with IAH, at increased risk of SHE, structured education programs have been shown to reduce severe hypoglycemia incidence and restore awareness in about 50% of patients [17]. A recent systematic review of interventions to restore awareness showed that these programs, characterized by 25–30 h of curriculum-based education delivered in groups over 1–5 weeks, demonstrated a halving of severe hypoglycemia rates [23]. These relatively cheap interventions have lasting effects and are cost effective [24]. For those who continue to have problematic hypoglycemia despite structured education, there are observational data demonstrating significant reductions in mild and severe hypoglycemia with CSII via insulin pump therapy [25, 26], borne out by meta-analysis showing a fourfold reduction in rates of severe hypoglycemia, with greatest reductions in those with highest baseline rates of hypoglycemia [27].

The advent of continuous glucose monitoring (CGM) that can alert and warn patients of impending hypoglycemia and, in particular, systems that integrate CGM data CSII systems capable of suspending insulin delivery in the presence of sensor-detected hypoglycemia have made a huge difference. In initial studies, those with high risk of severe hypoglycemia and IAH were excluded, which is why some of the earlier studies with CGM failed to show any reduction in hypoglycemia [28]. However, studies targeting those with IAH and using automated threshold suspend systems have shown significant reductions in severe hypoglycemia rates [29, 30]. The latest generation of CSII, the Medtronic 640G, can stop insulin delivery when it predicts hypoglycemia and has shown a reduction in hypoglycemia events [31]. Closed loop systems, that deliver insulin based on sensor glucose values, have been tested in short duration clinical trials for up to 3 months [32]. Some systems use insulin alone, but there are other systems in development that see both insulin and glucagon, more akin to a human pancreas, and theoretically better equipped to deal with problematic hypoglycemia. However, these “dual hormone” systems come with increased complexity, and currently available glucagon is not stable in solution for these to be commercially viable as yet. Most importantly, although both single and dual hormone systems improve glucose control and reduce hypoglycemia, these systems have not yet been tested in the high-risk IAH population.

The HypoCOMPASS trial randomized participants with IAH in a 2 × 2 algorithm to CSII or multi-dose insulin and CGM or self-monitoring of blood glucose. All patients were seen frequently over 6 months, and there was similar reduction in SHE from 8.9 to 0.8 events/year, with no difference between groups [33]. This study really highlights the importance of frequent contact and support to these patients. This evidence base for the approach to patients with problematic hypoglycemia was assimilated in a recent publication [34]. A recent publication demonstrated that following this algorithm, up to two thirds of patients with recurrent severe hypoglycemia can be managed conservatively, with resolution of severe hypoglycemia. However, islet transplantation can also deliver complete abolition of severe hypoglycemia with near-normalization of glucose control, albeit at the cost of risks of immunosuppression [35].

In one islet, center, over 70% of patients referred with severe recurrent problematic hypolycameia were able to resolve their issues with recurrent hypoglycaemia with conventional treatment alone [35].

#### Other Options

Pancreas and islet transplantations both offer treatment for IAH and severe hypoglycemia in patients with T1DM. Whole-organ pancreas transplants offer a higher rate of insulin independence compared to islets [36]; however, the procedure carries more risks and is associated with a higher complications rate [36] and is contraindicated in patients >60 years old and in patients and in those with a high cardiovascular risk. Therefore, in patients without these contraindications, there needs to be a discussion between the patient and the transplant team as to which would be the best option in each case.

Another important factor to take into consideration is the kidney function of the patient at the time of assessment. ICTs are contraindicated in patients with chronic kidney disease (i.e., eGFR <60 mL/min/1.73 m^2^) due to the risk of triggering end-stage renal failure by the use of immunosuppressive therapy. This group of patients often cause a clinical conundrum, requiring careful consideration of the pros and cons of risk of on-going severe hypoglycaemia versus the risks of accelerating any decline in renal function [37, 38]. Wherever possible, minimizing hypoglycaemia with the use of education and technology, with early liaison with renal teams to minimize any delays in listing for pre-emptive simultaneous islet or pancreas transplant with a kidney transplant would be the preferred option [34].

### Suitability for Islet Transplantation?

For most ICT programs, the indication is recurrent severe hypoglycemia despite optimal medical management as described above (Table 1). Inclusion criteria included the following: age 18–65 years, T1D for ≥5 years, absent stimulated C-peptide, IAH, and/or marked glycemic lability. If a patient meets those criteria, it is usually important to evaluate some other criteria, that identify those with likely optimal outcomes based on published data.

**Table 1:** Summary of indications and contraindications for islet cell transplantation

Indications for ICT
• Recurrent severe hypoglycaemia, including IAH or severe glycemic lability which is resistant to intensive insulin therapy
• Undetectable C-peptide levels (<0.3 ng/ml)
• 18–65 years old
• >5 years since diagnosis of T1DM
Absolute contraindications for ICT
• Detectable C-peptide levels (>0.3 ng/ml)
Relative contraindications for ICT
• Insulin requirements (>0.7 units/kg/day) or <15units/day
• HbA_1c_ >10%
• BMI >26 kg/m^2^ or weight <50 kg
• Creatinine >1.5 mg/dl and/or albuminuria >300 mg/24 h or measured GFR <80 ml/min/1.73 m^2^,
• Untreated arterial disease
• History of panel reactive anti-HLA antibodies
• Significant comorbidities

*ICT* islet cell transplantation, *IAH* impaired awareness of hypoglycaemia, *T1DM* type 1 diabetes mellitus, *BMI* body mass index, *HLA* human leukocyte antigen

For example, the CITR concluded that preservation of graft function (C-peptide ≥0.3 ng/mL) post final infusion is maximized by recipient age ≥35 years, baseline LDL<75, ≥500 K IEQs infused, use of Serva/NB1 collagenase, and calcineurin inhibitors. With these factors combined, the retention rate remains at 80% for 7–8 years. There is less agreement on the exclusion criteria, with significant differences among transplant programs; however, the most concurring contraindication is the presence of detectable C-peptide levels (>0.3 ng/ml) [39]. Considering the two international multicenter trials: In the Immune Tolerance Network, HbA1c >12%, BMI >26 kg/m^2^, insulin requirements >0.7 UI/kg/day, creatinine >1.5 mg/dl and/or albuminuria >300 mg/24 h, as well as the presence of infections or psychiatric diseases were considered exclusion criteria for transplantation [12, 37].

In the Clinical Islet Transplantation (CIT) Consortium study BMI >30 kg/m^2^, weight ≤50 kg, insulin requirement >1.0 UI/kg/day or <15 UI/day, HbA1c level >10%, measured GFR <80 ml/min/1.73 m^2^, history of panel-reactive anti-HLA antibodies by flow cytometry, and significant comorbid conditions were considered exclusion criteria for transplantation [15•] (Table 1).

## Conclusion

IAH and severe hypoglycemia have a huge impact on the quality of life of a substantial number of patients with T1DM. Following an evidence-based algorithm can resolve issues with severe hypoglycemia for a large proportion of patients and should be attempted. However, some patients continue to have problems with hypoglycemia despite these interventions, and for those current ICT offers complete resolution of SHE with near normalization of glucose control. The selection for the right candidate for this therapy is based on balancing the benefits (problematic hypoglycemia, improved glycemic control) against the risks (immunosuppression) for each individual case. Given the limited supply of organs for islet transplantation, it is important to select those who have most to benefit, and in whom other conventional strategies have been unsuccessful.
